# Supplementary material for: Candida albicans stimulates formation of a multi-receptor complex that mediates epithelial cell invasion during oropharyngeal infection
Source: PLoS Pathog. 2023 Aug 23;19(8):e1011579. doi: 10.1371/journal.ppat.1011579 (PMC10479894; doi:10.1371/journal.ppat.1011579)
Supplement: S1 Fig — (A) Immunofluorescent image of the OKF6/TERT-2 oral epithelial cell line infected with C. albicans and stained with control mouse IgG. Arrows indicate the organism in the magnified inset. Scale bar 10 μm. (B and C) Effects of knockdown of c-Met with siRNA (B) or inhibition of c-Met signaling with SGX523 (C) on the number of C. albicans cells that were associated (adherent and endocytosed) with the OKF6/TERT-2 oral epithelial cell line. (D) Immunoblot showing the effects of control and c-Met siRNA on the levels of the indicated oral epithelial cell proteins. (E) Effects of hepatocyte growth factor (HGF) treatment on the number of C. albicans cells that were associated with oral epithelial cells. (F) Immunoblot showing the phosphorylation of c-Met induced by a 20-min exposure to the indicated amounts of HGF. Results in (B, C, and E) are mean ± SD of 3 experiments performed in triplicate. ns, not significant (two-way Student’s t test [B and C] or one-way ANOVA with Sidak’s multiple comparisons test [E]). (PDF) [file ppat.1011579.s001.pdf]

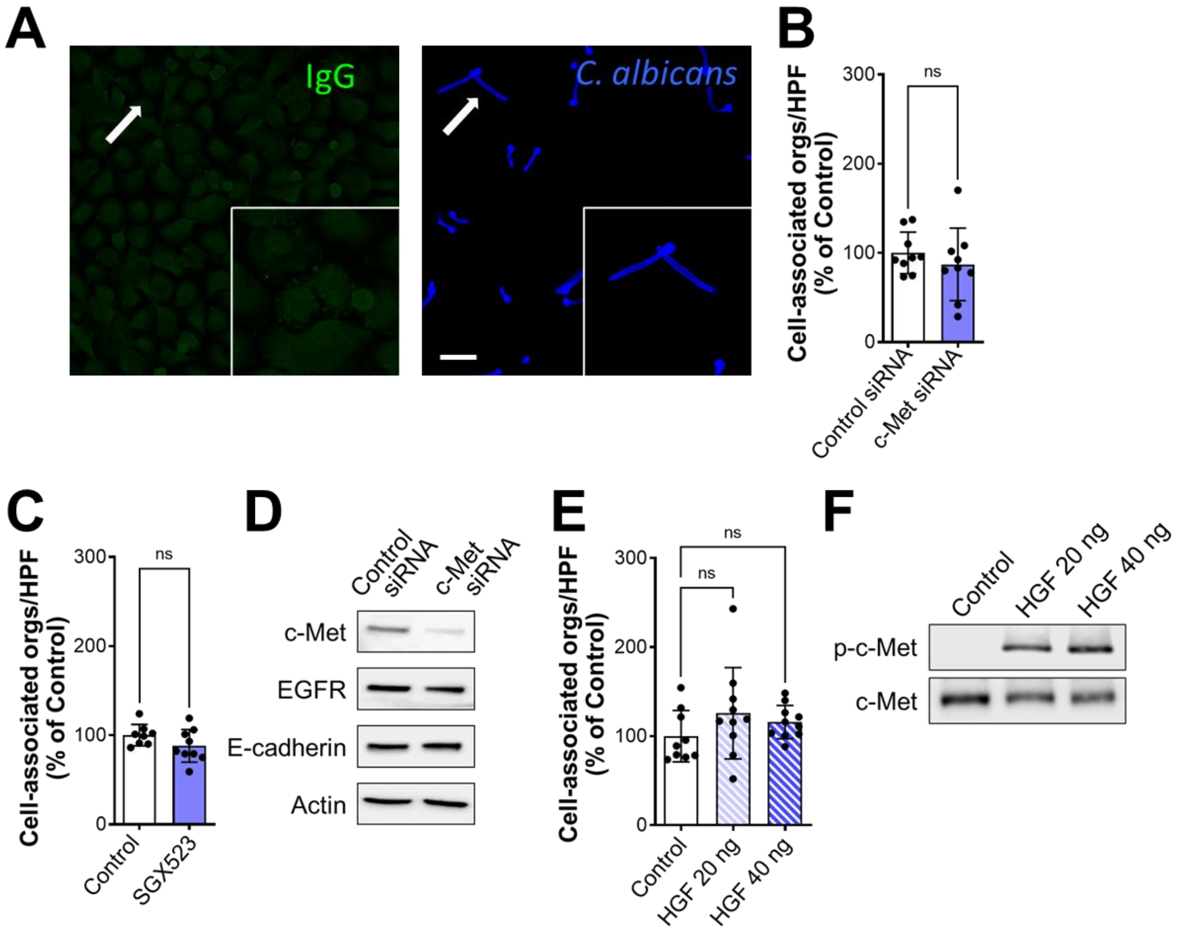

S1 Fig. (A) Immunofluorescent image of the OKF6/TERT-2 oral epithelial cell line infected with *C. albicans* and stained with control mouse IgG. Arrows indicate the organism in the magnified inset. Scale bar 10 µm. (B and C) Effects of knockdown of c-Met with siRNA (B) or inhibition of c-Met signaling with SGX523 (C) on the number of *C. albicans* cells that were associated (adherent and endocytosed) with the OKF6/TERT-2 oral epithelial cell line. (D) Immunoblot showing the effects of control and c-Met siRNA on the levels of the indicated oral epithelial cell proteins. (E) Effects of hepatocyte growth factor (HGF) treatment on the number of *C. albicans* cells that were associated with oral epithelial cells. (F) Immunoblot showing the phosphorylation of c-Met induced by a 20-min exposure to the indicated amounts of HGF. Results in (B, C, and E) are mean ± SD of 3 experiments performed in triplicate. ns, not significant (two-way Student's t test [B and C] or one-way ANOVA with Sidak's multiple comparisons test [E])
