# Supplementary material for: Candida albicans stimulates formation of a multi-receptor complex that mediates epithelial cell invasion during oropharyngeal infection
Source: PLoS Pathog. 2023 Aug 23;19(8):e1011579. doi: 10.1371/journal.ppat.1011579 (PMC10479894; doi:10.1371/journal.ppat.1011579)
Supplement: S2 Fig — (A and B) Effects of SGX523 and gefitinib on invasion (A) and adherence (B) of C. albicans to live and paraformaldehyde-fixed oral epithelial cells. (C) Effects of SGX523 and/or gefitinib on the number of C. albicans cells that were associated with oral epithelial cells. (D and E) The number of C. albicans cells that were associated with wild-type NIH/3T3 cells (control) or cells expressing human c-Met (D) or cells expressing the human epidermal growth factor (EGFR) and HER2 or human c-Met, EGFR, and HER2 (E). (F and G) Effects of siRNA knockdown of E-cadherin in combination with SGX523 (F) or gefitinib (G) on the number of C. albicans cells that were associated with oral epithelial cells. Results are mean ± SD of 3 experiments performed in triplicate. *p < 05, **p < 0.01, ***p, 0.001, ****p < 0.0001, ns; not significant (one-way ANOVA with Sidak’s multiple comparisons test [A-C, F, G] or two-tailed Student’s t test [D and E]). (PDF) [file ppat.1011579.s002.pdf]

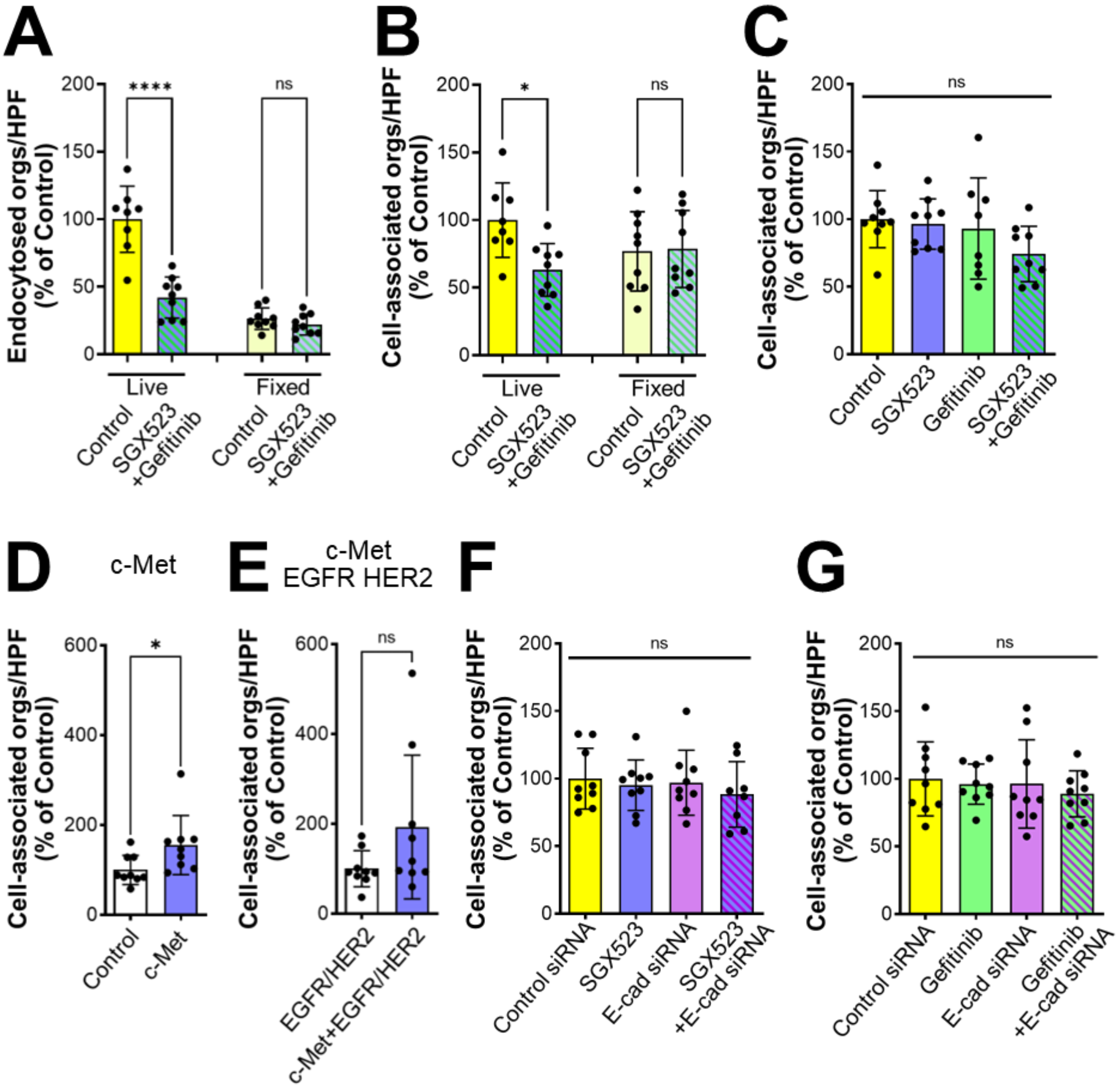

S2 Fig. (A and B) Effects of SGX523 and gefitinib on invasion (A) and adherence (B) of *C. albicans* to live and paraformaldehyde-fixed oral epithelial cells. (C) Effects of SGX523 and/or gefitinib on the number of *C. albicans* cells that were associated with oral epithelial cells. (D and E) The number of *C. albicans* cells that were associated with wild-type NIH/3T3 cells (control) or cells expressing human c-Met (D) or cells expressing the human epidermal growth factor (EGFR) and HER2 or human c-Met, EGFR, and HER2 (E). (F and G) Effects of siRNA knockdown of E-cadherin in combination with SGX523 (F) or gefitinib (G) on the number of *C. albicans* cells that were associated with oral epithelial cells. Results are mean ± SD of 3 experiments performed in triplicate. *p < 05, **p < 0.01, ***p ,0.001, ****p < 0.0001, ns; not significant (one-way ANOVA with Sidak's multiple comparisons test [A-C, F, G] or two-tailed Student's t test [D and E]).
