# Supplementary material for: Candida albicans stimulates formation of a multi-receptor complex that mediates epithelial cell invasion during oropharyngeal infection
Source: PLoS Pathog. 2023 Aug 23;19(8):e1011579. doi: 10.1371/journal.ppat.1011579 (PMC10479894; doi:10.1371/journal.ppat.1011579)
Supplement: S3 Fig — (A) Proximity ligation assay performed with mouse and rabbit IgG as a negative control. Scale bar 10 μm. (B-D) Co-immunoprecipitation experiments in oral epithelial cells transfected with control or E-cadherin siRNA and then infected with C. albicans for 20 min. Representative immunoblots of proteins obtained by immunoprecipitation with an anti-EGFR antibody (B). Densitometric analysis of 5 immunoblots (C and D). Results are mean ± SD. *p < 0.05, ***p < 0.001, ****p < 0.0001, ns; not significant (one-way ANOVA with Sidak’s multiple comparisons test). (PDF) [file ppat.1011579.s003.pdf]

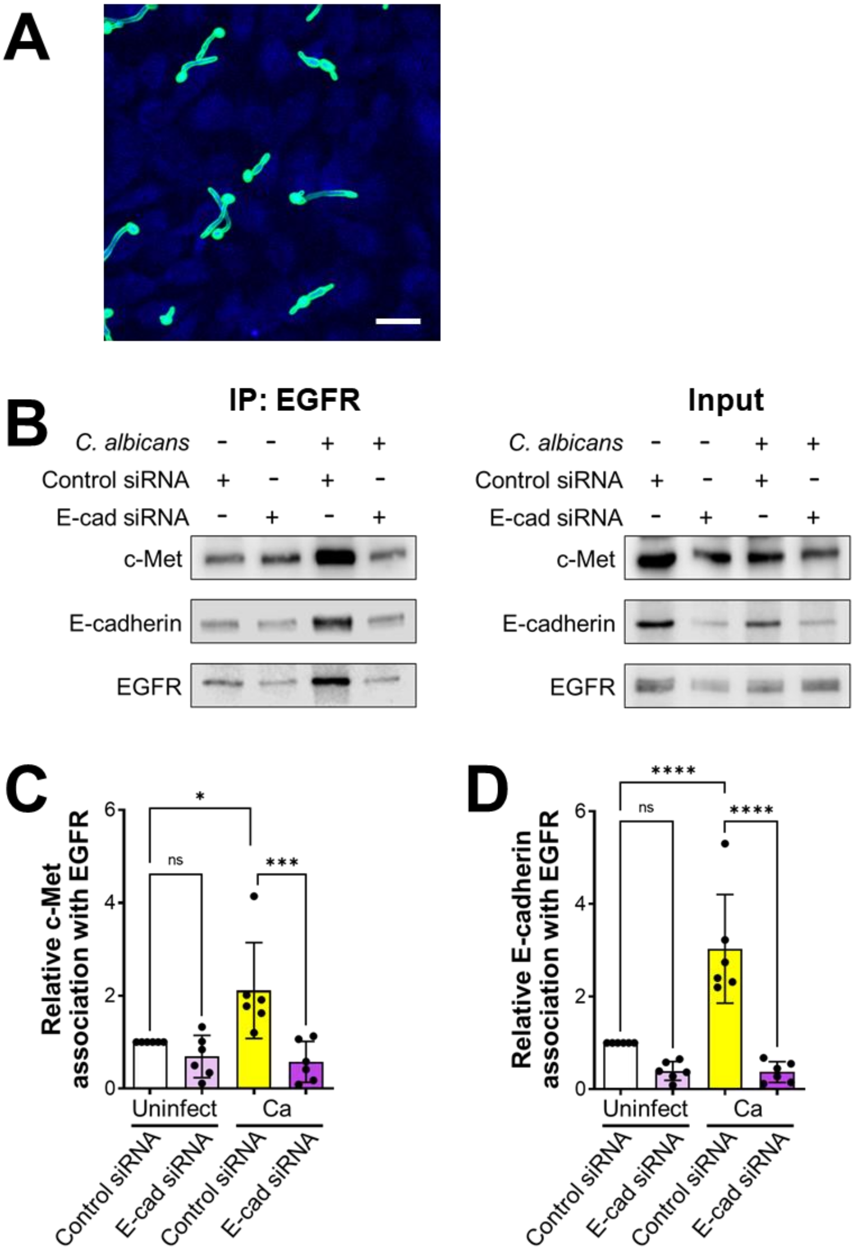

S3 Fig. (A) Proximity ligation assay performed with mouse and rabbit IgG as a negative control. Scale bar 10 µm. (B-D) Co-immunoprecipitation experiments in oral epithelial cells transfected with control or E-cadherin siRNA and then infected with *C. albicans* for 20 min. Representative immunoblots of proteins obtained by immunoprecipitation with an anti-EGFR antibody (B). Densitometric analysis of 5 immunoblots (C and D). Results are mean ± SD. *$p < 0.05$, ***$p < 0.001$, ****$p < 0.0001$, ns; not significant (one-way ANOVA with Sidak's multiple comparisons test).
