# Supplementary material for: Candida albicans stimulates formation of a multi-receptor complex that mediates epithelial cell invasion during oropharyngeal infection
Source: PLoS Pathog. 2023 Aug 23;19(8):e1011579. doi: 10.1371/journal.ppat.1011579 (PMC10479894; doi:10.1371/journal.ppat.1011579)
Supplement: S4 Fig — (A) Number of cells of the indicated C. albicans strains that were associated with oral epithelial cells. (B-D) Ece1 is dispensable for inducing the phosphorylation of c-Met but not EGFR in oral epithelial cells. Representative immunoblots (B). Densitometric analysis of 5 immunoblots showing the phosphorylation of c-Met (C) and EGFR (D) induced by the indicated strains of C. albicans. Results are mean ± SD. (E) Number of cells of the indicated C. albicans strains that were associated with oral epithelial cells. (F) Number of cells of the indicated C. albicans strains that were associated with live and paraformaldehyde fixed oral epithelial cells. Results in (A, E and F) are the mean ± SD of three experiments, each performed in triplicate. *p < 05, **p < 0.01, ****p < 0.0001, ns; not significant (one-way ANOVA with Sidak’s multiple comparisons test [B-D] or two-tailed Student’s t test [E and F]). (PDF) [file ppat.1011579.s004.pdf]

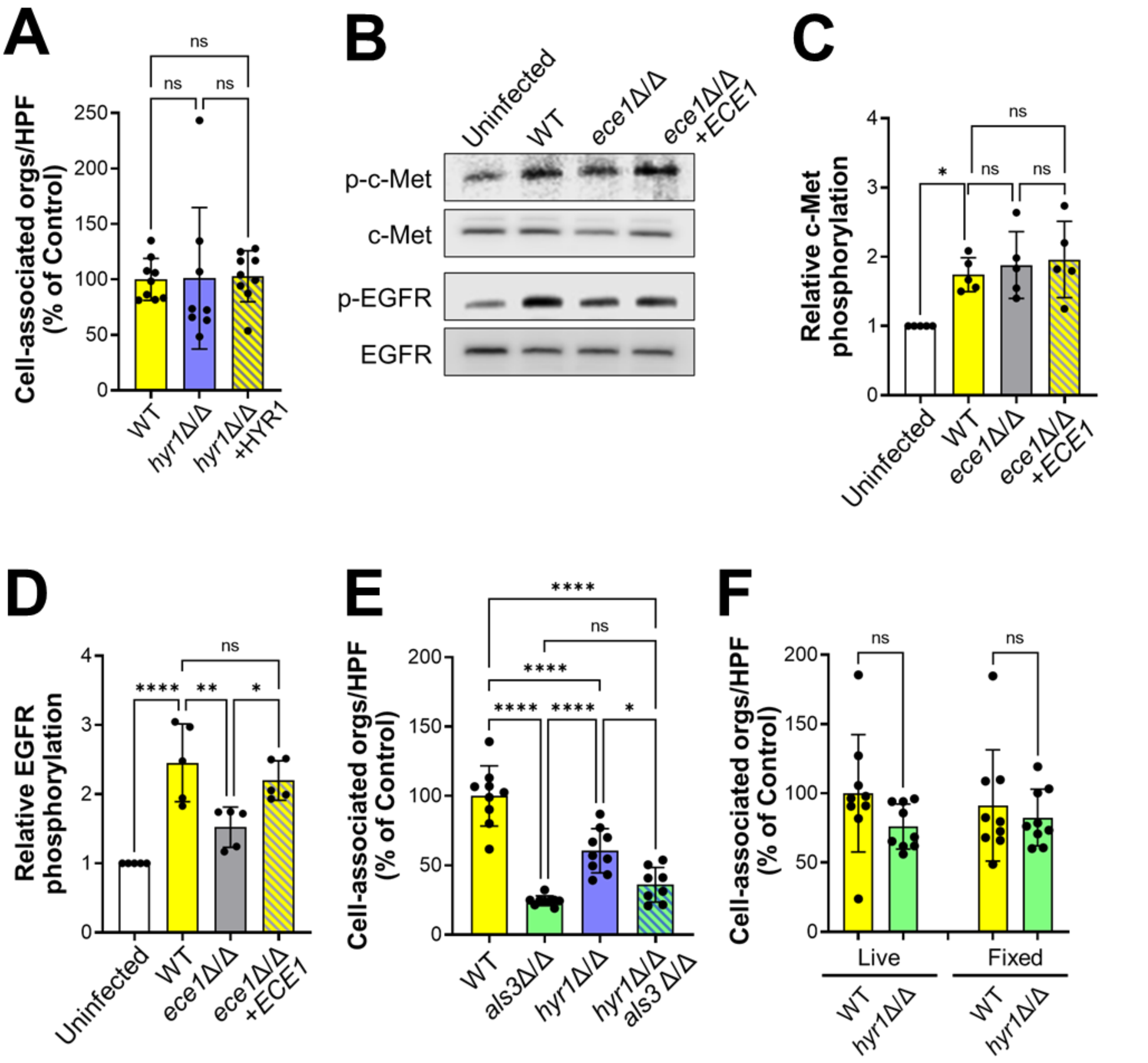

S4 Fig. (A) Number of cells of the indicated *C. albicans* strains that were associated with oral epithelial cells. (B-D) Ece1 is dispensable for inducing the phosphorylation of c-Met but not EGFR in oral epithelial cells. Representative immunoblots (B). Densitometric analysis of 5 immunoblots showing the phosphorylation of c-Met (C) and EGFR (D) induced by the indicated strains of *C. albicans*. Results are mean ± SD. (E) Number of cells of the indicated *C. albicans* strains that were associated with oral epithelial cells. (F) Number of cells of the indicated *C. albicans* strains that were associated with live and paraformaldehyde fixed oral epithelial cells. Results in (A, E and F) are the mean ± SD of three experiments, each performed in triplicate. *p < 05, **p < 0.01, ****p < 0.0001, ns; not significant (one-way ANOVA with Sidak's multiple comparisons test [B-D] or two-tailed Student's t test [E and F]).
