# Supplementary material for: Candida albicans stimulates formation of a multi-receptor complex that mediates epithelial cell invasion during oropharyngeal infection
Source: PLoS Pathog. 2023 Aug 23;19(8):e1011579. doi: 10.1371/journal.ppat.1011579 (PMC10479894; doi:10.1371/journal.ppat.1011579)
Supplement: S5 Fig — (A) Human neutrophils were infected with the indicated C. albicans strains constructed in the SN250 strain background. Results are mean ± SD of neutrophils from 5 donors, tested in triplicate. (B) Oral fungal burden of otherwise immunocompetent Mrp8;Metfl/fl mice, which have a neutrophil-specific deletion in c-Met (c-Met-/-) and their wild-type littermates after 2 days of infection with C. albicans SC5314. Results are combined data from 2 experiments with a mixture of male and female mice. ns, not significant; **p < 0.01 (two-sided Student’s t test). (PDF) [file ppat.1011579.s005.pdf]

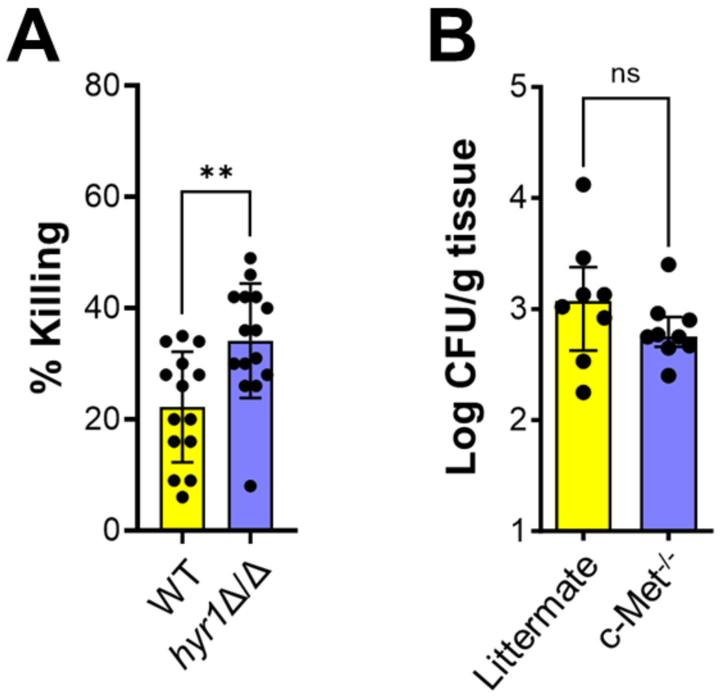

S5 Fig. (A) Human neutrophils were infected with the indicated *C. albicans* strains constructed in the SN250 strain background. Results are mean ± SD of neutrophils from 5 donors, tested in triplicate. (B) Oral fungal burden of otherwise immunocompetent Mrp8;*Met*$^{fl/fl}$ mice, which have a neutrophil-specific deletion in c-Met (c-Met$^{-/-}$) and their wild-type littermates after 2 days of infection with *C. albicans* SC5314. Results are combined data from 2 experiments with a mixture of male and female mice. ns, not significant; ***p* < 0.01 (two-sided Student's t test).
