# Supplementary material for: Candida albicans stimulates formation of a multi-receptor complex that mediates epithelial cell invasion during oropharyngeal infection
Source: PLoS Pathog. 2023 Aug 23;19(8):e1011579. doi: 10.1371/journal.ppat.1011579 (PMC10479894; doi:10.1371/journal.ppat.1011579)
Supplement: S6 Fig — The specimens were stained for c-Met, C. albicans, and DAPI (A) or EGFR, C. albicans, and DAPI (B). Epithelial cells are in the region above the dotted lines. Scale bar 50 μm. (PDF) [file ppat.1011579.s006.pdf]

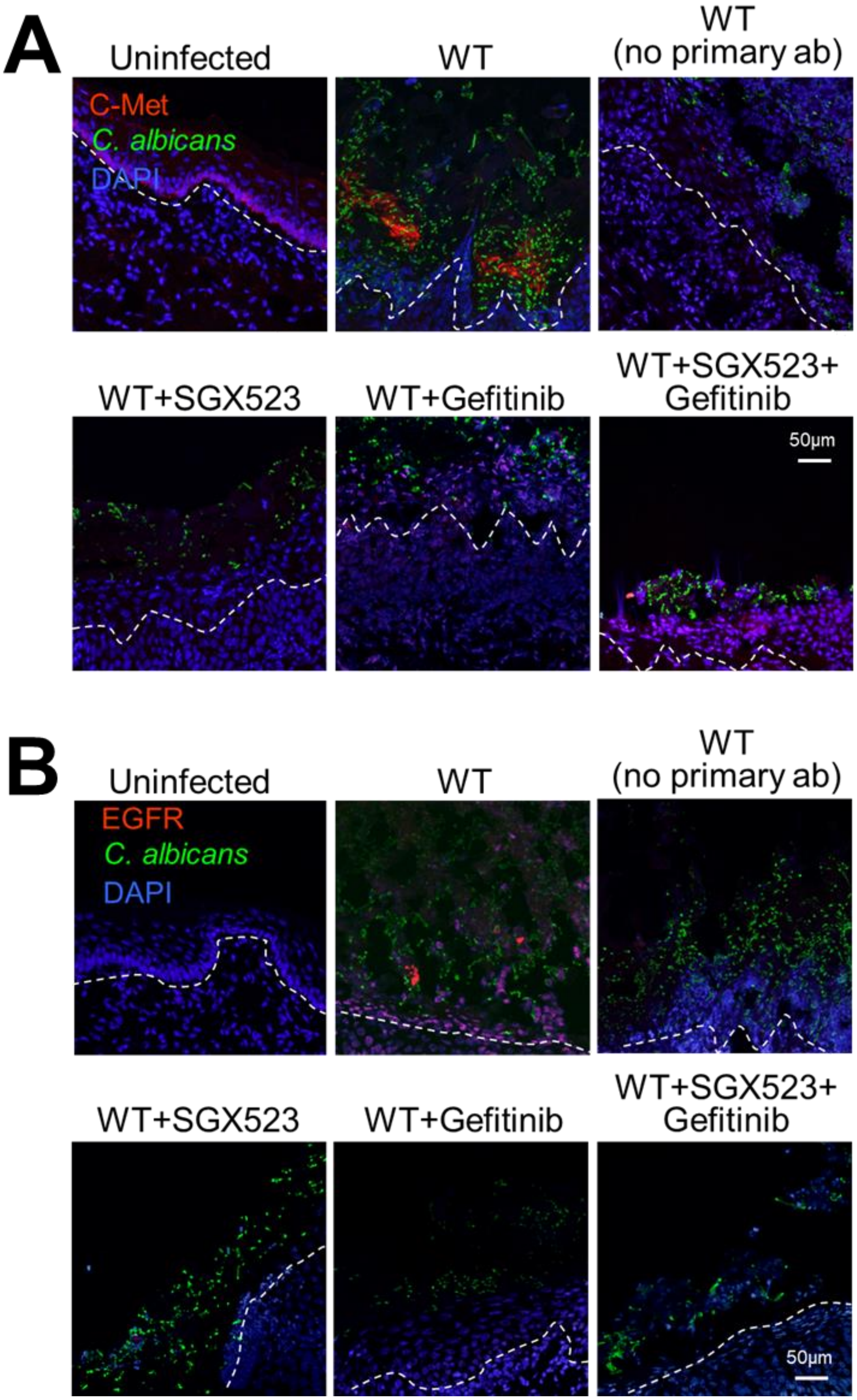

Fig. S6. Confocal micrographs of the tongues of mice immunosuppressed with cortisone acetate and then infected for 5 days with *C. albicans* strain SC5314 (WT) in the presence of SGX523 and/or gefitinib. The specimens were stained for c-Met, *C. albicans,* and DAPI (A) or EGFR, *C. albicans,* and DAPI (B). Epithelial cells are in the region above the dotted lines. Scale bar 50 μm
