# Supplementary material for: Candida albicans stimulates formation of a multi-receptor complex that mediates epithelial cell invasion during oropharyngeal infection
Source: PLoS Pathog. 2023 Aug 23;19(8):e1011579. doi: 10.1371/journal.ppat.1011579 (PMC10479894; doi:10.1371/journal.ppat.1011579)
Supplement: S2 Table — (PDF) [file ppat.1011579.s008.pdf]

S2 Table. List of *Candida albicans* strains used in the work.

Strain name	Relevant genotype	Reference
SC5314	Wild-type	[1]
SN250	*URA3/ura3Δ::λimm434 arg4Δ/arg4Δ his1Δ/his1Δ leu2Δ::CdHIS1/leu2Δ::CmLEU2*	[2]
CAI4 Clp10	*ura3Δ::λimm434/ura3Δ::λimm434 RPS10/rps10::URA3*	[3]
MLR63	*ura3Δ::λimm434/ura3Δ::λimm434 ARG4::URA3::arg4::hisG/arg4::hisG his1::hisG::pTEF1-GFP/his1::hisG*	[4]
JL036	*als3Δ::frt/als3Δ::frt*	[5]
MC355	*hyr1Δ::r1CdHIS1r1, his1Δ::r3*	This work
MC374	*hyr1Δ::r1CdHIS1r1/hyr1Δ::r1CdHIS1r1 his1Δ::r3/his1Δ::r3 als3Δ::r3NAT1r3/als3Δ::r3NAT1r3*	This work
MC502	*hyr1Δ::r1CdHIS1r1/hyr1Δ::r1CdHIS1r1 his1Δ::r3/his1Δ::r3, mdr1Δ::NAT1-HYR1/mdr1Δ::NAT1-HYR1*	This work
823	*URA3/ura3Δ::λimm434 arg4Δ/arg4Δ his1Δ/his1Δ IRO1/iro1Δ::λimm434 hyr1Δ::CdHIS1/hyr1Δ::CmLEU2*	[6]
ssa1/als3	*ura3Δ::λimm434 ssa1::FRT ssa2::FRT als3 rps10::SSA1-URA3 ura3Δ::λimm434 ssa1::FRT SSA2 als3::NAT1 RPS10*	[7]
JL018	*ece1Δ::frt/ece1Δ::frt*	[5]

1. Jones T, Federspiel NA, Chibana H, Dungan J, Kalman S, Magee BB, et al. The diploid genome sequence of *Candida albicans*. Proc Natl Acad Sci USA. 2004;101(19):7329-34. PubMed PMID: 15123810.
2. Noble SM, Johnson AD. Strains and strategies for large-scale gene deletion studies of the diploid human fungal pathogen *Candida albicans*. Eukaryot Cell. 2005;4(2):298-309. Epub 2005/02/11. doi: 10.1128/EC.4.2.298-309.2005. PubMed PMID: 15701792; PubMed Central PMCID: PMCPMC549318.
3. Brand A, MacCallum DM, Brown AJ, Gow NA, Odds FC. Ectopic Expression of *URA3* can influence the virulence phenotypes and proteome of *Candida albicans* but can be overcome by targeted reintegration of *URA3* at the *RPS10* locus. Eukaryot Cell. 2004;3(4):900-9. PubMed PMID: 15302823.
4. Richard ML, Nobile CJ, Bruno VM, Mitchell AP. Candida albicans biofilm-defective mutants. Eukaryot Cell. 2005;4(8):1493-502. PubMed PMID: 16087754.
5. Swidergall M, Solis NV, Millet N, Huang MY, Lin J, Phan QT, et al. Activation of EphA2-EGFR signaling in oral epithelial cells by *Candida albicans* virulence factors. PLoS Pathog. 2021;17(1):e1009221. Epub 2021/01/21. doi: 10.1371/journal.ppat.1009221. PubMed PMID: 33471869; PubMed Central PMCID: PMCPMC7850503.
6. Noble SM, French S, Kohn LA, Chen V, Johnson AD. Systematic screens of a *Candida albicans* homozygous deletion library decouple morphogenetic switching and pathogenicity. Nat Genet. 2010;42(7):590-8. Epub 2010/06/15. doi: 10.1038/ng.605. PubMed PMID: 20543849; PubMed Central PMCID: PMCPMC2893244.
7. Liu Y, Mittal R, Solis NV, Prasadarao NV, Filler SG. Mechanisms of *Candida albicans* trafficking to the brain. PLoS Pathog. 2011;7(10):e1002305. Epub 2011/10/15. doi: 10.1371/journal.ppat.1002305PPATHOGENS-D-11-01220 [pii]. PubMed PMID: 21998592; PubMed Central PMCID: PMC3188548.
