# Supplementary material for: Candida albicans stimulates formation of a multi-receptor complex that mediates epithelial cell invasion during oropharyngeal infection
Source: PLoS Pathog. 2023 Aug 23;19(8):e1011579. doi: 10.1371/journal.ppat.1011579 (PMC10479894; doi:10.1371/journal.ppat.1011579)
Supplement: Attachment — Submitted filename: C-met Hyr1 paper - rebuttal.pdf [file ppat.1011579.s010.pdf]

General comments:

Reviewer #1:
Comment 1. Overall, this is an important contribution to the field, with some places where a few additional experiments would increase the author's ability to make these conclusions.

Response. Thank you.

Reviewer #2:
Comment 1. However, it is not clear why neutrophil c-Met would be expected to be involved in this interaction as it is unknown whether it functions in a similar manner to epithelial c-Met.

Response. It is known that c-Met is expressed on neutrophils and that it can alter neutrophil activity against cancer cells (see ref. 30), but whether c-Met can influence the anti-candidal activity of neutrophils was previously unknown. Using neutrophils that were deficient in c-Met, we found that although this receptor is required for maximal neutrophil killing of C. albicans in vitro, it does not appear to interact with Hyr1. These data are presented in Fig. 8A.

Comment 2. The asl3/hyr1 double mutant CFUs are assessed in neutropenic mice. The rationale for this experiment is not clear since neutropenic mice will still have some neutrophils with activity.

Response. Although there may have been some minor residual neutrophil activity in mice treated with the anti-GR-1 antibody, comparison of the oral fungal burden of the control mice with the mice that received the anti-GR-1 antibody demonstrates these neutrophils had minimal activity against C. albicans (Fig. 10C and D). At day 1 post-infection with the WT strain, the mice treated with the anti-GR-1 antibody had a 1-log greater oral fungal burden than the control mice, and at day 2 post-infection, this difference increased to 2.5-log. Because the anti-GR-1 antibody substantially reduced the activity of phagocytes against C. albicans, we feel that the conclusions from experiments with this antibody are justified.

Comment 3. The double mutant has very slightly fewer CFU than WT so premature to conclude that the differences in CFUs are a result of differences in phagocyte killing.

Response. We assume that the reviewer is referring to the day 1 timepoint (Fig. 10C). In the control (phagocyte-replete) mice, the *hyr1*Δ/Δ *als3*Δ/Δ mutant achieved a significantly lower oral fungal burden than the WT strain. This difference was about 0.5-log and was very similar to that seen in the initial experiments with this mutant, which were repeated twice (Fig. 10A). Thus, in 3 independent experiments, mice infected with the *hyr1*Δ/Δ *als3*Δ/Δ double mutant had a lower fungal burden than mice infected with the WT control strain. When the mice were treated with the GR-1 antibody to deplete phagocytes, the oral fungal burden achieved by the *hyr1*Δ/Δ *als3*Δ/Δ mutant was similar to the WT strain. We believe that this absence of a virulence defect of the double mutant in the phagocyte-deficient mice suggest that in phagocyte-replete mice, the reduced virulence of this mutant is due mainly to its increased susceptibility to phagocyte killing. We have modified this paragraph (lines 240-244) to hopefully convey this conclusion more clearly.

Comment 4. Similarly mice treated with dual inhibitors of EGFR and c-Met have slightly reduced CFU (is this with WT C. albicans?) that don't correspond with the histopathology sections. Since animals are ingesting systemic drugs, it is not clear how specific the effects are to oral epithelium.

Response. The data with the combined inhibitors represent the results of two independent experiments. The mice treated with the combined inhibitors had >5-fold lower oral fungal burden than mice control mice. In the figure legend, we have clarified that these results were obtained from mice infected with the WT strain. We agree that the c-Met and EGFR inhibitors likely blocked receptor signaling in multiple types of host cells, which is the reason why we concluded that "the combined inhibition of EGFR and c-Met ameliorated the severity of OPC" without mentioning what the potential target cells were (lines 335-336).

Reviewer #3:

Comment 1. In Figure 1F, can the authors show or provide a reference that adding the ligand HGF increases the levels or phosphorylation of c-Met?

Response. As suggested by the reviewer, we have done immunoblotting with a phosphospecific c-Met antibody to demonstrate that HGF induces c-Met phosphorylation (Fig. S1F).

Comment 2. In Figure 9, the authors nicely show that inhibition of both c-Met and EGFR is required in vivo for a phenotype in infected mice. Since the authors have showed data earlier in their paper on conditional KO mice for c-Met in neutrophils, if the authors still have this colony of mice available, it would be worth infecting them in the OPC model to examine CFUs post-infection after steroids or even in immunocompetent mice. One may expect that these mice won't have a phenotype given the pharmacological inhibition data of Fig 9 but sometimes there are effects that can be revealed with genetic manipulation at the cell specific level that may be masked or missed otherwise. If the mice are not available anymore, the authors could discuss these experiments (alongside conditional KO mice for c-Met in epithelium crossed with Krt-13 or Krt-14 mice) in the discussion.

Response. We have added oral fungal burden data comparing the mice with neutrophil-specific deletion of c-Met with their wild-type littermates. There was no difference in oral fungal burden after 2 days of infection in these otherwise immunocompetent mice (Fig. 5B).

Part II – Major Issues: Key Experiments Required for Acceptance
Please use this section to detail the key new experiments or modifications of existing

Reviewer #1: Major points:
Comment 1. (Figure 1) it looks like phospho-c-Met peaks at 10 minutes, but the quantification shows an increase over the entire timepoint—is this really a representative blot?

Response. When the densitometric data were normalized to total c-Met, there was a consistent increase in c-Met phosphorylation over time. However, to make this increase clearer to the reader, we have replaced this blot with another one.

Comment 2. (Figure 2) It would be nice to see in Figure 2E whether c-Met, when transfected alone, also is phosphorylated in response to Candida challenge. It is important to know whether c-Met is activatable, not just whether endocytosis occurs.

Response. To address this issue, we stained infected NIH/3T3 cells that expressed no human receptors, human c-Met, human EGFR/HER2, or human c-Met and EGFR/HER2 with C. albicans. We then stained the cells with a phosphospecific anti-c-Met antibody and imaged them by confocal microscopy. As expected, no c-Met phosphorylation was detected in control

NIH/3T3 cells that expressed either no human receptors or human EGFR and HER2 (Fig. 2G). C. albicans infection induced modest c-Met phosphorylation in NIH/3T3 cells that expressed human c-Met alone. By contrast, C. albicans induced strong c-Met phosphorylation in NIH/3T3 cells that expressed human c-Met, EGFR, and HER2. Thus, the presence of EGFR and HER2 is required for C. albicans to induce maximal phosphorylation of c-Met.

Comment 3. (Figure 4) The authors start by talking about Ssa1, but then it is dropped. It might be worth re-writing to either focus only on the Als3, or add additional data on the role of Ssa1.

Response. We agree with this suggestion and have removed Ssa1 from the figure (now Fig. 6). We also explicitly mentioned that we focused our investigations on Hyr1 and Als3 (line 142).

Comment 4. The title of Figure 4 is that Hyr1 interacts with c-Met and EGFR, but the data in this figure doesn't explicitly show Hyr1-EGFR interaction, only Hyr1-cMet.

Response. As suggested, we deleted EGFR from the title of the figure (now Fig. 5).

Comment 5. (Figure 5) The model shows that the three fungal proteins are needed to coordinate the complex formation of the three host receptors. The authors should include immunofluorescence experiments, like in Figure 1, showing that the host receptors no longer co-localize when the fungal proteins are deleted. The authors should also show that the co-localization does not occur when one of the host receptors is deleted/depleted.

Response. While we agree that the suggested experiments would be interesting, we respectfully point out that the current manuscript is quite long with 1 table, 10 figures, 6 supplementary figures, and 3 supplementary tables. We believe that performing these experiments should be the topic of a subsequent manuscript.

Comment 6. (Figure 8) although the text says there is no difference in CFU at the early time points, the immunofluorescence shows a decrease in anti-Candida staining in the mutant strains. Potentially there is some clearance that is beyond the resolution of the CFU analysis? This should be discussed.

Response. There is substantial lesion-to-lesion variation in the number of fungal elements that are visible by immunofluorescence. We strongly feel that the CFUs provide a more accurate assessment of oral fungal burden in the mice.

Reviewer #2:
Comment 1. Co-Ip experiments were convincing using hyphal Candida cells, however is the same association found with yeast from cells? This specificity is shown for phosphorylation, but is it also true for protein association?

Response. We only performed the Co-Ip experiments with hyphal cells. Because yeast phase organisms do not stimulate either c-Met or EGFR, we speculate that they will not stimulate the formation of a multi-receptor complex.

Comment 2. If Hyr1 like Asl3 protects from neutrophil killing and in epithelial cells is involved in endocytosis, it is very likely that this protect in neutrophils is a result of decreased phagocytosis. A phagocytic experiment would add to the understanding of the basis for its function in neutrophils.

Response. The reviewer may be correct that Hyr1 and Als3 reduce phagocytosis. However, it is also possible that they the decrease production of reactive oxygen species. We respectfully suggest that performing experiments to determine the mechanisms by Hyr1 and Als3 reduce neutrophil killing of C. albicans is beyond the scope of the current manuscript.

Comment 3. The animal experiments are not compelling is associating c-Met or EGFR with infection levels of tested hyphal proteins. As animals are ingesting systemic drugs, it is not clear how specific the effects are to oral epithelium- can this be shown on tongues as done for Fig. 8?

Response. To demonstrate that the inhibitors reduced c-Met and EGFR phosphorylation in the infected oral tissues, we treated immunosuppressed mice with SGX523, gefitinib, or both inhibitors. After 5 days of infection, we sacrificed the mice, harvested the tongues, and assessed the phosphorylation of c-Met and EGFR by indirect immunofluorescence with phosphospecific antibodies. Although there was substantial epithelial cell destruction at this late time point, we found that treatment with both SGX523 and gefitinib reduced expression of c-Met and EGFR (Fig. S6).

Reviewer #3: (No Response)

Part III – Minor Issues: Editorial and Data Presentation Modifications

Reviewer #1: Minor points:
Comment 1. (Figure 1) please include uninfected control images. Please also show representative activation of cMET in response to HGF.

Response. We have done this (Fig. S1A and F).

Comment 2. Supplemental figure 2B and C are inconsistent between the data, the text, and the significance values shown on the graph. The text says that 'neither inhibitor reduced C. albicans adherence' but the authors show a statistically significant difference.

Response. We thank the reviewer for pointing this out. The combination of SGX523 and gefitinib modestly inhibited C. albicans adherence in some experiments (Fig. S2B), but not others (Fig. S2C). We have added this information to the text (lines 64-65).

Comment 3. (Figure 2I) the bimodal phosphorylation of EGFR in the uninfected cells is potentially interesting and should be commented on.

Response. We are not sure what bimodal phosphorylation of EGFR the reviewer is referring to. This figure demonstrates that C. albicans infection induces EGFR phosphorylation in epithelial cells treated with control siRNA whereas infection of epithelial cells in which E-cadherin has been knocked down induces less EGFR phosphorylation (Fig 3A-C). These results are described in lines 88-90.

Comment 4. (Figure 5) typo in the middle—Als3 vs. Als31

Response. Corrected.

Comment 5. Figure 6C) asterisks are cropped

Response. Corrected.

Comment 6. Typo line 202 ‘interacting of with’

Response. Corrected.

Reviewer #2:

Comment 1. Fig. 8 A and B, -please add some marking on images indicating where basement membrane is to delineate epithelium from connective tissues to orient reader to where c-Met and EFGR are being expressed.

Response. We have done so.
